# Influence of the Body Schema on Multisensory Integration: Evidence from the Mirror Box Illusion

**DOI:** 10.1038/s41598-017-04797-0

**Published:** 2017-07-11

**Authors:** Yuqi Liu, Jared Medina

**Affiliations:** Department of Psychological and Brain Sciences, University of Delaware, 105 The Green, Room 108, Newark, DE 19716 USA

## Abstract

When placing one hand on each side of a mirror and making synchronous bimanual movements, the mirror-reflected hand feels like one’s own hand that is hidden behind the mirror. We developed a novel mirror box illusion to investigate whether motoric, but not spatial, visuomotor congruence is sufficient for inducing multisensory integration, and importantly, if biomechanical constraints encoded in the body schema influence multisensory integration. Participants placed their hands in a mirror box in opposite postures (palm up, palm down), creating a conflict between visual and proprioceptive feedback for the hand behind the mirror. After synchronous bimanual hand movements in which the viewed and felt movements were motorically congruent but spatially in the opposite direction, participants felt that the hand behind the mirror rotated or completely flipped towards matching the hand reflection (illusory displacement), indicating facilitation of multisensory integration by motoric visuomotor congruence alone. Some wrist rotations are more difficult due to biomechanical constraints. We predicted that these biomechanical constraints would influence illusion effectiveness, even though the illusion does not involve actual limb movement. As predicted, illusory displacement increased as biomechanical constraints and angular disparity decreased, providing evidence that biomechanical constraints are processed in multisensory integration.

## Introduction

To perceive our bodies and the world around us, our brain needs to efficiently and accurately integrate inputs from different modalities into a coherent representation. It has been well established that unimodal sensory inputs are weighted in proportion to each input’s precision in multisensory integration–the optimal weighting principle. For example, when spatial position information from vision and proprioception are dissociated by viewing through a prism, participants often estimate their hand to be closer to the visual estimate–a modality with higher spatial precision compared to proprioception^1–3^. Weighting based on sensory error is statistically optimal in that it maximizes accuracy and minimizes variance in estimations^2, 4–6^. This pragmatic value has made optimal weighting a central principle in a variety of computational models on multisensory integration^7–9^.

In addition to optimal weighting from sensory inputs, prior knowledge accumulated with experience can also provide information for solving the problem of multisensory integration^8, 9^. This is especially likely with the body, an object for which we have a lifetime of prior knowledge. A number of studies have proposed the existence of the body schema, an online representation of body position in space^10–12^. Encoded in the body schema is information regarding resistance from muscles and joints during movements that determines the allowable range and physical difficulty of motion–what we will refer to as biomechanical constraints^13, 14^. For example, supination of the right hand from a pronated (palm facing down) to a supinated (palm facing up) posture encounters resistance at the end of the rotation. This movement is more biomechanically constrained compared to the opposite movement, pronation of the right hand from a palm up to a palm down position. Importantly, these biomechanical constraints influence not only physical movements, but are instantiated in body representations. When asked to judge whether a hand image presented at different orientations is a left or right hand, reaction times increased as the orientation of the viewed hand differed from the participant’s own hand posture^13–16^. Reaction times for trials with the same orientation displacement between the viewed hand and the participant’s own hand were longer for the more biomechanically constrained rotation direction, providing evidence that participants utilized a body schema that encodes biomechanical constraints to make chirality judgments^17–19^.

Studies of the rubber hand illusion have provided some evidence that biomechanical constraints can influence multisensory integration and ownership of the fake hand. In the rubber hand illusion, when individuals see a life-sized rubber hand stroked synchronously with their hidden actual hand, they often perceive the rubber hand as their own (ownership illusion) along with perceiving their hand as closer to the rubber hand (proprioceptive shift)^20^. Critically, the rubber hand illusion decreased when the rubber hand was placed at an anatomically implausible posture^21–23^, also see ref. 24 (e.g. fingers pointing towards the body) or was distant from the body^25, 26^, suggesting that anatomical features about one’s body contributes to the effectiveness of this illusion. In one study, this illusion was examined with the rubber hand rotated to one of eight different positions^27^ (0° to 315° relative to the actual hand). The rubber hand illusion was more effective when it was placed at angles that could be more easily reached by the actual hand, providing more detailed evidence that multisensory integration and body ownership are influenced by anatomical plausibility (i.e. the range of movements). Other studies also demonstrated that visual and tactile information from the fake hand and actual unseen hand respectively became less integrated when the fake hand was at an anatomically implausible versus plausible posture^28, 29^. Taken together, these studies indicate that anatomical plausibility of the viewed hand posture plays an important role in multisensory integration^30, 31^.

Another method used to examine multisensory integration and body ownership is the mirror box illusion. In this illusion, an individual places a hand on each side of a mirror aligned with the participant’s midsagittal plane. When looking into the mirror, a reflection of the hand in front of the mirror (mirror hand) looks like the unseen hand behind the mirror^32^. A spatial conflict between visual and proprioceptive inputs can be introduced by moving the unseen hand to a position that is incongruent with the mirror hand^33–38^. During this conflict, individuals typically perceive their hand as closer to the visual estimate, often reporting that their hand is located where they see it^35–38^. These results have been explained using optimal weighting principles–as vision is more accurate than proprioception, participants are more likely to report their hand closer to the visual estimate.

Although the rubber hand illusion is an important paradigm, it is limited in that the rubber hand lacks verisimilitude, and is typically static (though see ref. 39 for an exception). However, one can manipulate visuomotor synchrony in the mirror box illusion by moving both hands either synchronously or asynchronously^36–38^. When making synchronous movements (e.g. tapping the index finger), movements of the mirror-reflected hand and the actual hand are both motorically congruent (i.e. joints flex and extend synchronously) and spatially congruent (e.g. fingers moving in the same direction in space). Given the addition of motor congruence, and that the mirror-reflected hand looks more like the participant’s own hand (compared to a rubber hand), this paradigm can create a robust proprioceptive shift and sense of illusory hand ownership^38^. Furthermore, one can manipulate various aspects of the illusion to understand multisensory integration and body ownership. In this manuscript, we developed a novel variant of the mirror box illusion to answer two questions: (1) Is *motoric* visuomotor congruence alone, even with incongruence in an external frame of reference, sufficient for inducing multisensory integration and body ownership and (2) Does information from the body schema, specifically biomechanical constraints, contribute to multisensory integration and body ownership?

**Figure 1:**
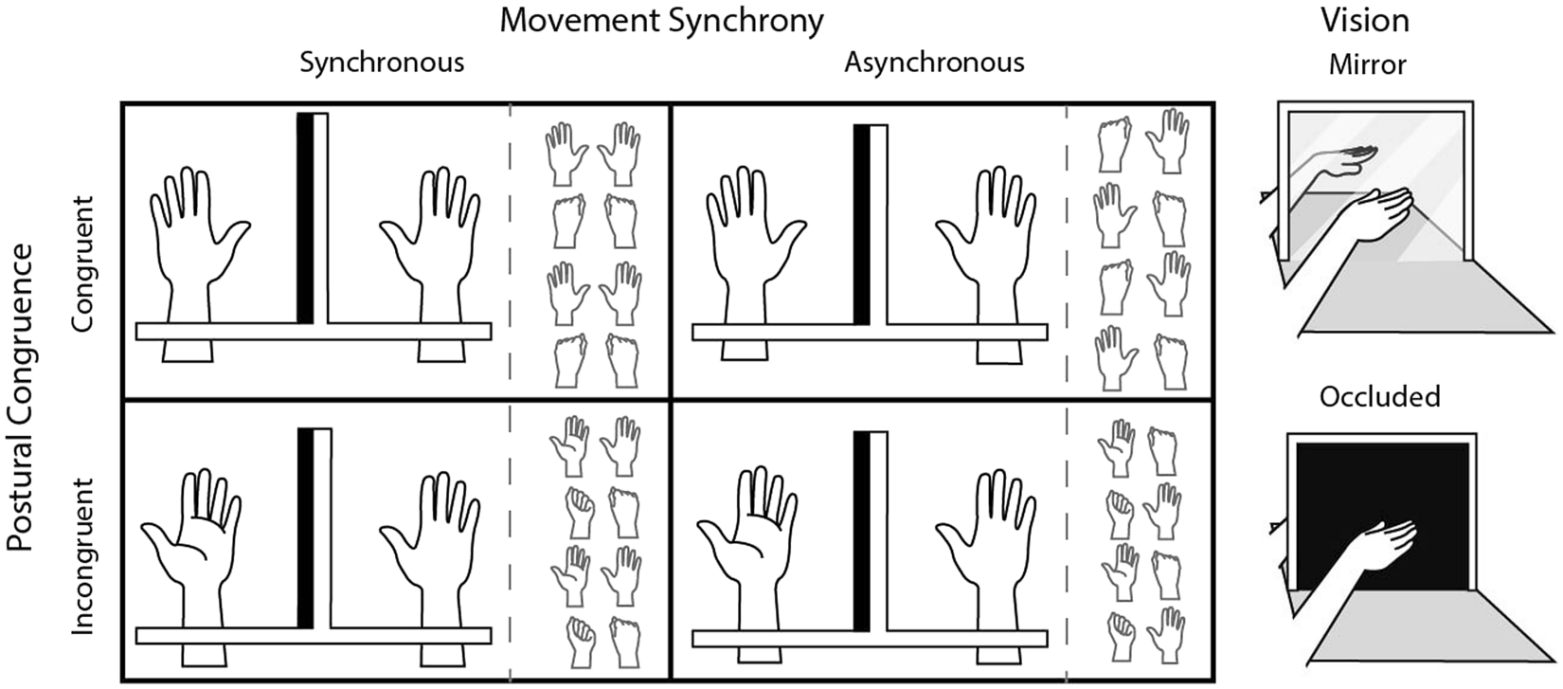
Top-down view of hand postures and movement (left) and vision conditions (right) in Experiment 1. Left: Each box represents a trial type in the mirror vision condition. Participants placed their left hand behind the mirror and the right hand in front of the mirror, with hands in either congruent (top) or incongruent (bottom) postures. Hand position, shown at each half beat of the metronome (starting at top) is shown on the right side of each box. Movements were either synchronous (left) or asynchronous (right). Right: Participants viewed the reflection of their right hand in the mirror vision condition (top) and a black sheet in the occluded vision condition (bottom).

To address the first question, we altered the traditional mirror box illusion and introduced a different source of spatial conflict–hand posture. In Experiment 1, participants placed their right hand in front of the mirror and their left hand behind the mirror. The hands were placed either in the same (palm down; Fig. 1, upper left boxes), or opposite postures (palm up versus palm down; Fig. 1, lower left boxes). A variant of Luria’s bimanual coordination task was used^40–42^, in which participants synchronously or asynchronously opened and closed their hands in the mirror box (see Fig. 1). In the critical condition, the hands were in incongruent postures but moved synchronously (see Fig. 1, lower left box), such that the viewed and felt movements were motorically congruent, but incongruent in external space. If motoric congruence alone could drive multisensory integration and body ownership, we predict that motorically synchronous movements would facilitate multisensory integration and body ownership even with spatial incongruence. A prior study used a mental rotation task to implicitly measure the perceived hidden hand posture when the rubber hand and actual hand were in opposite postures^43^, here we explicitly measured the perceived hidden hand posture as an index of multisensory integration. Based on the optimal weighting principle, participants would feel the hidden hand in or near the same posture as the visual estimate (palm down), as well as feel a sense of ownership of the viewed hand. Consistent with our prediction, participants reported that the felt posture of the unseen left hand “flipped” to either the exact posture of the mirror hand or to an orientation rotated towards the mirror hand posture.

**Figure 2:**
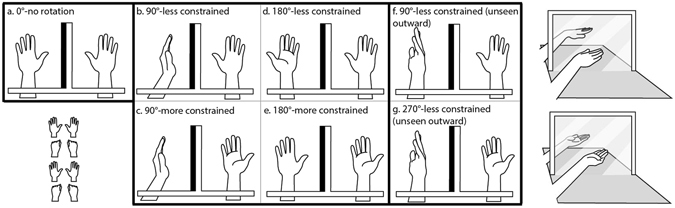
Top-down view of hand postures in Experiment 2 with the left hand behind the mirror (**a**–**g**). The mirror reflections of the hand are shown in the top and bottom right panel. An example sequence of hand movements is shown in the bottom left panel, in which participants opened and closed both hands synchronously. Each condition is named based on the amount of angular disparity defined as the difference (in degrees) between the proprioceptively-defined and visually-defined hand posture, and (within each angular disparity group) whether the rotation between the two hand postures is more or less biomechanically constrained. The “outward” posture in conditions f and g means palm facing away the body midline, reached by clockwise rotation of the left hand (and counter-clockwise rotation of the right hand) by 90° from the palm-down posture.

Using this new illusion, we then examined our second question–whether biomechanical constraints from the body schema influences the amount of illusory displacement. If so, we predicted more illusory displacement when the rotation from the proprioceptively defined posture (i.e. the actual unseen hand posture) to the visually defined posture (i.e. the posture of the mirror-reflected hand) was shorter (e.g. 90° versus 180°) as well as less biomechanically constrained. To test this hypothesis, we measured illusory displacement while manipulating angular disparity and the amount of biomechanical constraints between the proprioceptively and visually defined hand postures, shown in Fig. 2. The conditions are named based on the amount of angular disparity and whether the rotation between the two hand postures is more or less biomechanically constrained. Consistent with our hypothesis, we found illusory displacement decreased as the angular disparity and biomechanical constraints between the actual and viewed hand posture increased, providing evidence that information regarding biomechanical constraints contributes to multisensory integration.

Along with reporting perceived limb position, a second variable used in assessing body illusions is ownership - whether the mirror or rubber hand feels like the participant’s own hand. Although some research has shown a strong relationship between changes in perceived limb position and a sense of ownership^20, 27, 38, 44^, more recent studies have found that changes in perceived limb position can occur without changes in perceived ownership^23, 45, 46^, providing evidence for a dissociation between ownership and changes in perceived limb position. Given this, we also examined whether participants’ sense of body ownership changed based on this illusion.

## Results

**Figure 3:**
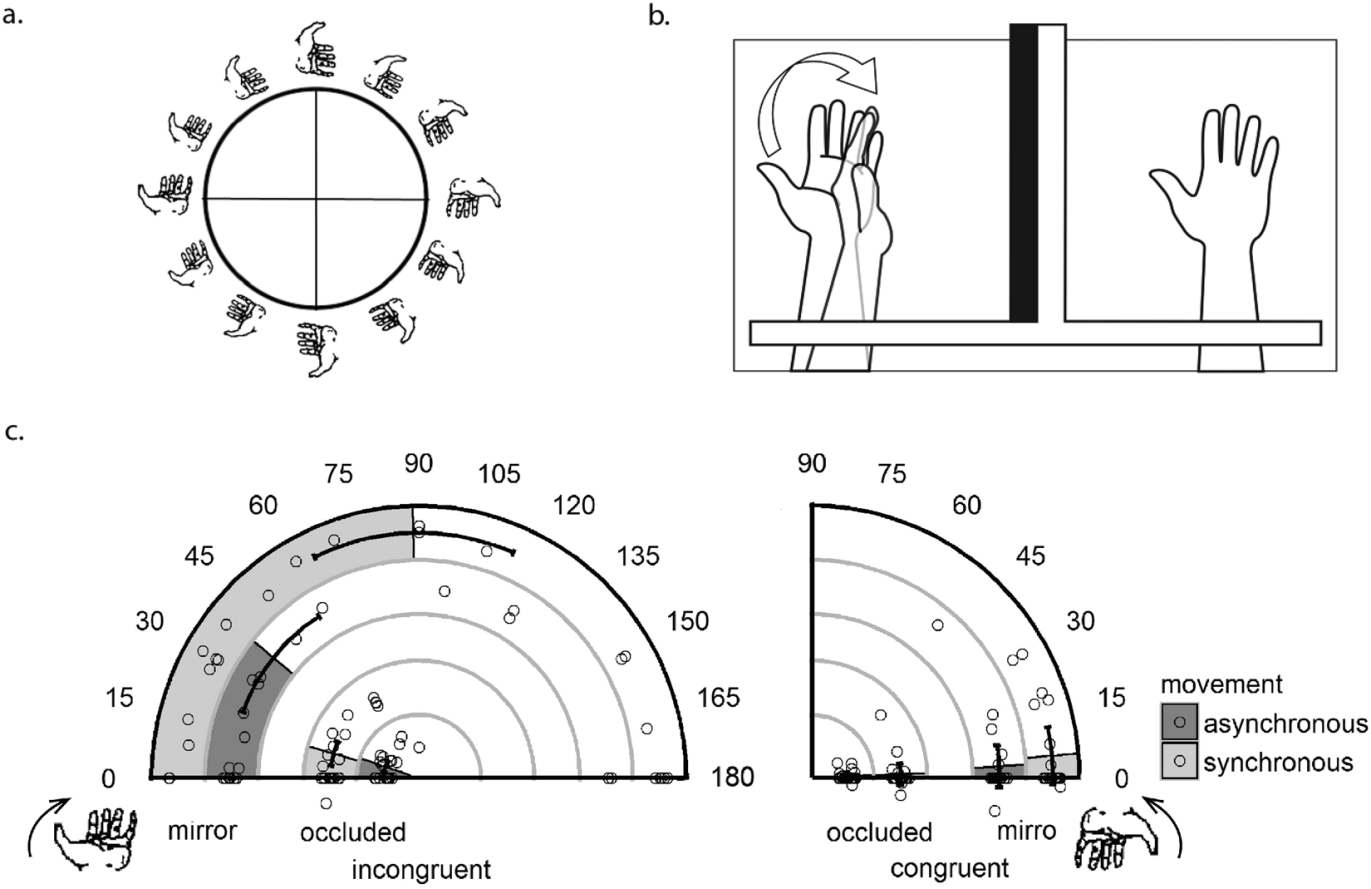
(**a**) The circular posture scale used in Experiments 1 and 2. Participants were asked to mark on this circle the perceived position of their hidden hand. (**b**) Mean postural displacement in degrees in Experiment 1, circles represent individual data points. Zero indicates no postural displacement (relative to the proprioceptively-defined posture of their hidden hand), whereas 180 indicates participants reporting their hands in the opposite posture (matching what they saw in the mirror condition). In the incongruent condition, the unseen (left) hand was palm up and the right hand was palm down, whereas in the congruent condition, both hands were in the same posture (palm down). Whenever shown, error bars indicate 95% within-subjects confidence intervals^52^. As illusory displacement was minimal for congruent trials, we plotted from 0° to 90° for more efficient display. (**c**) An artistic rendering of postural displacement in Experiment 1. When the viewed hand reflection was palm down and the actual hand was palm up, some individuals perceived their hand as having moved towards the visual estimate.

In this section, we report changes in the perceived posture of the unseen (left) hand and sense of ownership of the mirror-reflected hand. Changes in the perceived posture of the unseen (left) hand were measured using a circular posture scale (Fig. 3a). From this measure came our dependent variable, postural displacement, defined as the difference in degrees between the participant’s response on the circular posture scale and the actual posture of their unseen left hand. We also measured participants’ sense of ownership (see Table S1 in Supplemental Materials for how it was calculated), and subjective ratings of unseen (left) hand posture (results in Supplemental Materials).

### Experiment 1

In this experiment, we examined the effect of hand posture, movement congruence, and mirror viewing on perceived hand position and ownership. Here, both hands were either in the same or opposite postures (see Fig. 1).

### Postural Displacement

For all trials, positive displacement denotes changes in the biomechanically less constrained direction (clockwise when the left palm was up, counter-clockwise when the left palm was down). Note that in the incongruent condition, positive displacement indicated illusory displacement towards the palm down mirror hand.

As shown in Fig. 3b (left), there was substantially greater postural displacement in incongruent trials with mirror vision compared to other conditions, which led to a main effect of postural congruence (incongruent: *M* = 41.06°, *SD* = 28.44°; congruent: *M* = 3.54°, *SD* = 7.23°), *p* < 0.001, as well as a significant vision by postural congruence interaction, *p* < 0.001. Specifically, the difference in postural displacement between incongruent and congruent trials with mirror vision (incongruent: *M* = 65.19°, *SD* = 52.06°; congruent: *M* = 4.38°, *SD* = 9.58°) was substantially greater than in the occluded vision (incongruent: *M* = 16.94°, *SD* = 21.90°; congruent: *M* = 2.71°, *SD* = 6.87°) condition, providing evidence that visual information led to illusory displacement of perceived hidden hand posture (see Fig. 3c). Note that even in the occluded vision condition, there was more postural displacement in the incongruent (unseen left hand palm up) versus congruent (unseen left hand palm down) condition, *p* < 0.001. One possibility is that when the hand was in a more biomechanically constrained posture (i.e. palm up), variance in proprioceptive estimation increased and led to a bias towards the less biomechanically constrained postures.

We also found a main effect of movement synchrony, as synchronous movements (*M* = 28.67°, *SD* = 17.42°) resulted in greater postural displacement than asynchronous movements (*M* = 15.94°, *SD* = 16.74°), *p* = 0.006. Interactions between movement synchrony and vision, *p* = 0.008, along with movement synchrony and postural congruence, *p* = 0.004, were significant. Most importantly, a significant three-way interaction between postural congruence, movement synchrony, and vision was observed, *p* = 0.008. There was significant postural displacement when the actual and viewed hand posture was incongruent, but only when the hands were making synchronous movements and in the mirror vision condition.

### Sense of ownership

**Figure 4:**
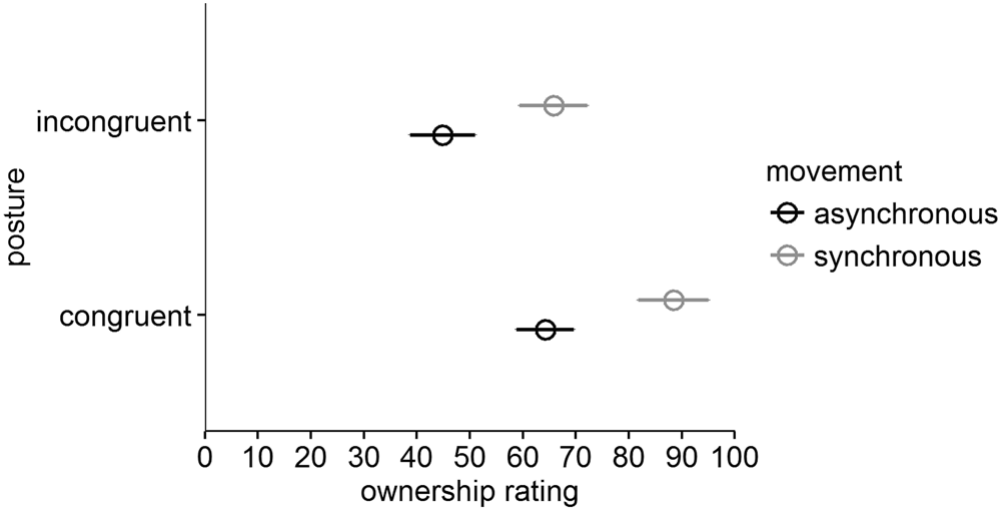
Mean ownership ratings in Experiment 1. Higher ratings indicate that the viewed hand was more strongly felt as the viewer’s own hand.

Given that there was no “mirror hand” in the no mirror condition, ownership ratings were collected only for the mirror viewing condition. Ownership ratings were consistent with perceived postural displacement (Fig. 4). As expected, we found main effects of postural congruence, *p* < 0.001 (congruent: *M* = 76.37, *SD* = 17.03, incongruent: *M* = 55.33, *SD* = 25.91), and movement synchrony, *p* < 0.001 (synchronous: *M* = 77.13, *SD* = 20.17, asynchronous: *M* = 54.57, *SD* = 24.06), with no interaction between these factors, *p* = 0.660.

We performed additional analyses to demonstrate that motoric congruence influences perceived body ownership. First, in the congruent condition, with the viewed and hidden hands in the same posture, visual and proprioceptive information were identical only in the synchronous movement condition. Given this, we found the expected result–participants had higher ownership ratings in the synchronous (*M* = 88.47, *SD* = 15.88) versus asynchronous (*M* = 64.27, *SD* = 25.45) condition, *p* < 0.001. In the incongruent posture, visual information regarding the hand did not match proprioceptive information in *both* the synchronous and asynchronous conditions. If motoric congruence contributes to a sense of ownership, regardless of spatial incongruence, we would predict higher ownership ratings in the synchronous versus asynchronous conditions. We found this to be the case (synchronous: *M* = 65.79, *SD* = 30.45, asynchronous: *M* = 44.88, *SD* = 27.64), *p* = 0.009, demonstrating that motoric congruence also contributes to a sense of ownership.

To examine the relationship between sense of ownership and postural displacement, we ran Spearman’s correlation tests between ownership ratings and postural displacement in incongruent posture trials (unseen hand palm up, mirror-reflected hand palm down). A significant correlation was found in both the synchronous (*ρ* = 0.72, p < 0.001) and asynchronous conditions (*ρ* = 0.60, *p* = 0.002).

In summary, we found the greatest postural displacement and highest ownership ratings when the mirror and unseen hand were in congruent postures and making synchronous movements. These results are consistent with previous findings regarding the spatial and temporal rule of multisensory integration^47, 48^. Most importantly, visual inputs combined with *motorically* synchronous movements resulted in illusory displacement of perceived hand posture and higher ownership ratings, even in conditions with large postural discrepancies between visually- and proprioceptively-defined body postures. This provides evidence that motoric congruence alone could induce multisensory integration and an increased sense of ownership of the mirror hand.

### Experiment 2

To investigate the effect of biomechanical constraints on multisensory integration, we used the same paradigm as Experiment 1, but manipulated the rotational distance (i.e. angular displacement) and biomechanical constraints between the hidden hand posture and mirror hand posture. To examine if our findings generalize to both hands, we examined whether having the left or right hand hidden behind the mirror influenced performance.

### Postural displacement

We present results related to the biomechanical constraints hypothesis in the main text (performance in the 0° condition is presented in the Supplemental Material). To compare illusory displacement across conditions with different amounts of angular disparity, we used postural displacement percentage (postural displacement divided by angular disparity) as our dependent variable. Positive postural displacement percentage indicates biases towards the visual estimate, and negative values indicates biases away from the visual estimate.

**Figure 5:**
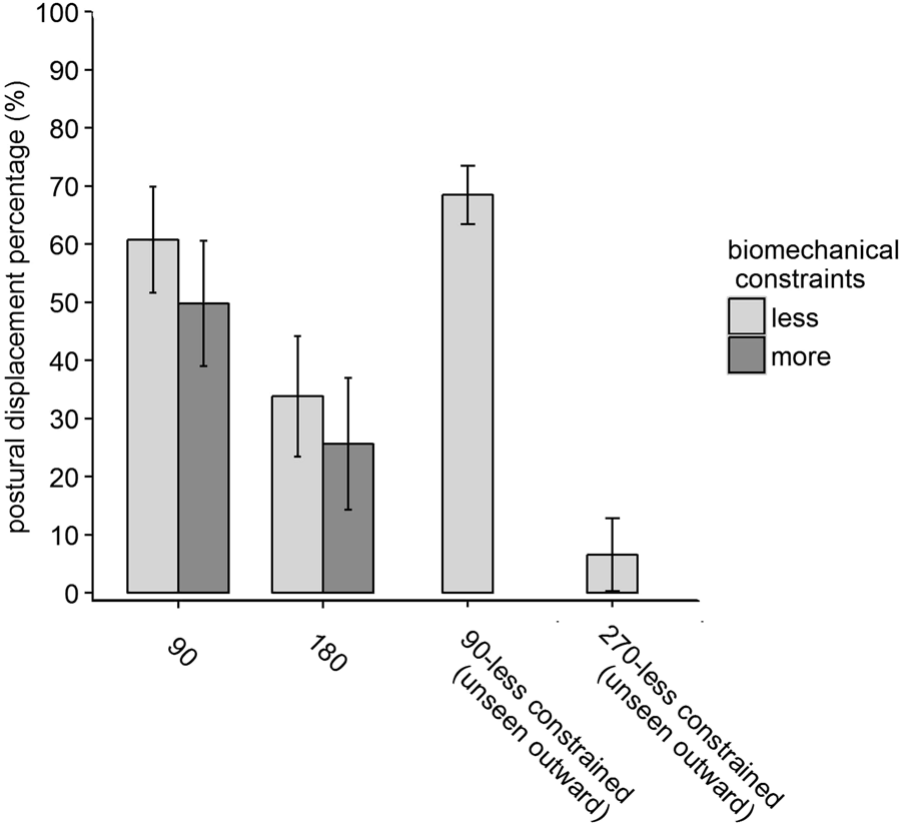
Mean postural displacement percentage in Experiment 2, collapsed across the left and right hidden hand.

To test the hypothesis that illusory displacement would increase with decreased angular disparity and decreased biomechanical constraints between the proprioceptive and visual estimate, we ran a permutation test with angular disparity (90° and 180°) and biomechanical constraints (less and more) as within-subject factors, and hidden hand (left and right hand) as between-subjects factor (see Fig. 5). To control for hidden and viewed hand posture, only the conditions in Fig. 2b–e were included in this analysis as conditions 2b and 2c were matched for hidden hand posture, and conditions 2d and 2e were both with hands palm up and palm down. As expected, we found a main effect of angular disparity, *p* < 0.001, as the postural displacement percentage was greater for the 90° (*M* = 55.34%, *SD* = 29.44%) than the 180° condition (*M* = 29.73%, *SD* = 29.66%). Importantly, there was a main effect of biomechanical constraints, *p* = 0.032, with greater postural displacement percentage for less (*M* = 47.34%, *SD* = 26.38%) versus more (*M* = 37.72%, *SD* = 31.98%) biomechanically constrained conditions. No interactions were significant (*p*s > 0.25). Consistent with our hypothesis, information from the body schema is processed in multisensory integration, such that multisensory integration, indexed by postural displacement percentage, increased as angular disparity and biomechanical constraints between the proprioceptive and visual estimate decreased.

Next, we compared performance in the 90°-less constrained (unseen outward, Fig. 2f) and the 270°-less constrained (unseen outward, Fig. 2g) conditions to examine whether biomechanical constraints influenced illusory displacement even for relatively long rotations (see Fig. 5). In the 270°-less constrained (unseen outward) condition, the absolute angular disparity is 90°. However, to match the posture of the viewed hand, the actual hand would have to rotate 270° given biomechanical constraints. If multisensory integration was constrained by biomechanical constraints, angular disparity would be perceived as 270° instead of 90°, resulting in less integration than the 90°-less constrained (unseen outward) condition. Otherwise, angular disparity would be perceived as 90° and one would predict similar integration as in other 90° conditions.

First, illusory displacement in the 270°-less constrained (unseen outward) condition was in the biomechanically plausible direction for more than 75% of participants, indicating the effects of biomechanical constraints on perception. Although some individuals demonstrated displacement in the less biomechanically plausible direction, those displacement percentages were small (*M* = *−*14.48%, *SD* = 9.46%) and likely reflected estimation variance in trials with no illusory displacement. We did a permutation test with condition (270°- and 90°-less constrained, unseen outward) as the within-subjects factor and hidden hand (left and right) as the between-subjects factor. There was a main effect of condition, *p* < 0.001, with a significantly smaller postural displacement percentage in the 270°- (*M* = 6.54%, *SD* = 21.98%) than 90°-less constrained (unseen outward) condition (*M* = 68.47%, *SD* = 34.65%). Therefore, although the absolute angular disparity was 90° in the 270°-less constrained (unseen outward) condition, it was likely perceived as 270° as an effect of biomechanical constraints. Compared with the 90°-less constrained (unseen outward) condition, longer angular disparity in the 270°-less constrained (unseen outward) condition resulted in significantly less multisensory integration. The condition by hidden hand interaction was not significant, *p* = 0.96.

### Sense of ownership

**Figure 6:**
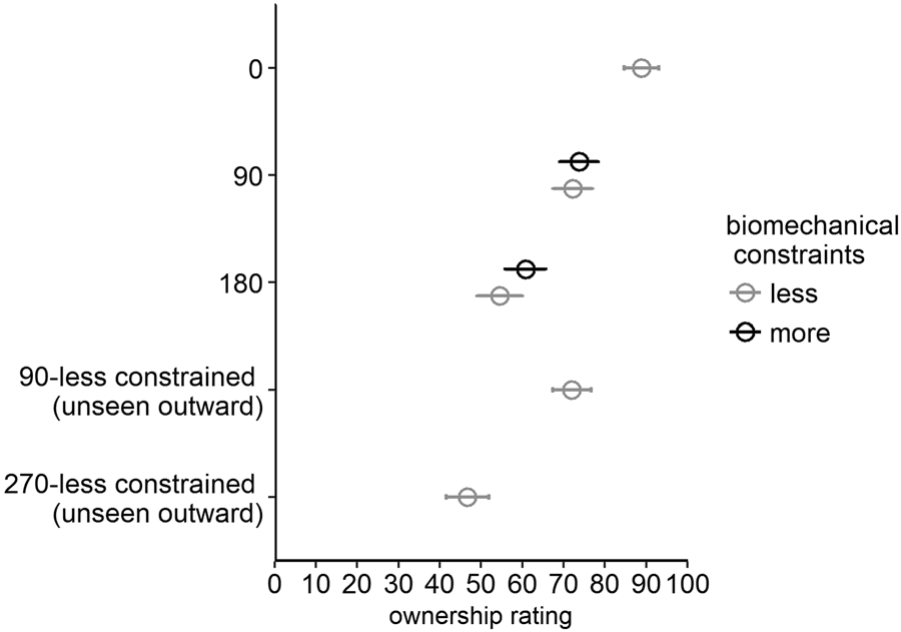
Mean ownership ratings in Experiment 2, collapsed across the left and right hand.

We ran a permutation analysis with angular disparity (90° and 180°) and biomechanical constraints (less, more) as within-subjects factors, and hidden hand (left, right) as the between-subjects factor (see Fig. 6). There was a significant main effect of angular disparity, *p* < 0.001, as there was a higher ownership rating in the 90° (*M* = 72.98, *SD* = 18.17) versus 180° conditions (*M* = 57.66, *SD* = 23.67). However, contrasting our findings with postural displacement percentage, we did not find a significant main effect of biomechanical constraints, *p* = 0.23 (less: *M* = 63.38, *SD* = 20.49; more: *M* = 67.25, *SD* = 20.15). All interactions were not significant (*p*s > 0.20).

We then compared ratings in 90°-less constrained and 270°-less constrained (unseen outward) conditions to examine whether biomechanical constraints were taken into account in perceiving angular disparity (see Fig. 6). We ran a permutation test with condition (90°-less constrained and 270°-less constrained, unseen outward) as the within-subjects factor and hidden hand (left and right hand) as the between-subjects factor. Consistent with the postural displacement results, ratings were significantly higher in the 90°-less constrained (*M* = 71.99, *SD* = 23.76) than the 270°-less constrained (unseen outward) condition (*M* = 46.70, *SD* = 26.87), *p* < 0.001, indicating that sense of ownership can be influenced by biomechanical constraints. There was no interaction between condition and hidden hand, *p* = 0.70.

As in Experiment 1, we ran a Spearman correlation analysis between ownership ratings and postural displacement percentage for each condition (see Table 1). Given no main effect of hidden hand chirality (left, right) on postural displacement percentage and ownership ratings, we collapsed data over hidden hand. Ownership ratings were significantly correlated with postural displacement percentage in all but the 270° condition–the condition with the least postural displacement and lowest ownership ratings.

**Table 1:** Correlation between ownership ratings and postural displacement percentage in Experiment 2.

Angular disparity	90	90	180	180	90	270
Biomechanical constraints	less	more	less	more	Less (unseen outward)	Less (unseen outward)
Spearman correlation coefficient (*ρ*)	0.55	0.76	0.34	0.39	0.54	0.01
*p*-value	<0.001	<0.001	0.018	0.007	<0.001	0.94

In summary, we found that both postural displacement percentage and sense of ownership increased as the angular disparity from the proprioceptively-defined to visually-defined posture decreased. Importantly, postural displacement percentage also decreased with increased biomechanical constraints. In addition, there was substantially less postural displacement and lower ownership ratings in the 270°- versus 90°- less constrained conditions, which matched in absolute angular disparity but differed in biomechanical constraints. These findings provided evidence that when the visual and proprioceptive hand are in incongruent postures, information from the body schema contributes to multisensory integration.

### General discussion

Our study demonstrated two novel findings. First, we showed that motoric congruence alone is sufficient for inducing multisensory integration and changes in perceived ownership. When moving the hands synchronously while in opposing postures in the mirror box, the viewed and felt movements were motorically congruent, but were spatially in the opposite direction. Nevertheless, participants perceived illusory displacement of the unseen hand posture towards the visual estimate of the mirror hand, and felt increased ownership over the mirror hand. These findings indicate that congruence in a motor reference frame can strongly influence multisensory integration and perceived body ownership. Second, our results provide novel evidence that additional information–apart from unimodal sensory precision–constraints multisensory integration. Specifically, the amount of illusory displacement was predicted by the difficulty in rotating between the unseen and viewed hand. In Experiment 2, illusory displacement increased as the rotation between the proprioceptively defined to visually defined hand posture became shorter and less biomechanically constrained.

Our findings add to prior evidence that the rubber hand illusion is abolished when placed in an anatomically implausible position^21–23, 27^. First, whereas prior studies focused on the effect of angular disparity between the rubber hand and the actual hand on multisensory integration^27^, we found multisensory integration affected by biomechanical constraints (i.e. rotation direction), even when the angular disparity was the same. Second, prior studies found the effect of biomechanical constraints on ownership illusion and proprioceptive shift, whereas we demonstrated the effect on a different dimension of body representation–hand posture, indicating that biomechanical constraints might be a common mechanism underlying multiple aspects of body representation.

What mechanism can explain the observed changes in postural displacement and ownership? Recently, a causal inference model has been developed to explain multisensory integration and body ownership^30, 49, 50^. In the causal inference model, the system first estimates the probability that information from different modalities comes from a common cause or independent causes before integrating or segregating information. In our illusion in the incongruent posture (e.g. seen hand palms-down, hidden hand palms-up), participants are initially well aware that visual and proprioceptive information is conflicting. Therefore, the initial assumption is likely that the information from different modalities is from independent causes, such that the viewed hand is simply a reflection, and that the actual hand is in the opposite posture. In the rubber hand illusion, as congruent information is presented across modalities (e.g. synchronous visuotactile stimulation), evidence builds in support of a common cause and then leads to a sense of ownership over the rubber hand. In our illusion, no participants immediately felt the hand rotate, nor immediately reported ownership over the mirror hand. The illusion occurred only after some period of time with congruent hand movements, consistent with work demonstrating that the accumulation of evidence is involved in making inferences regarding common or independent causes^25, 27, 30^. The perceived common cause then drives the integration of sensory inputs from different modalities, leading to postural displacement and changes in the sense of ownership.

Models explaining the rubber hand and other body ownership illusions have posited a number of factors that contribute to the inference of whether a viewed hand and one’s own hand represent a common cause. For example, it is well established that as information from different modalities becomes more temporally and spatially congruent, it is more likely to be perceived as coming from a common source^47, 48^. Furthermore, congruence of other body-specific factors such as hand chirality and shape, can lead to changes in perceived body ownership (see ref. 30 for a review). Our findings add to these models, indicating that two novel sources of information also contribute to deciding on a common cause. First, previous studies assume an external frame of reference when referring to spatial cross-modal congruence^47^. Our results show that motoric congruence can lead to inferring that the mirror and hidden hand come from a common cause (i.e. the mirror-reflected hand is my hand), even in the face of postural and spatial visuomotor incongruence. One possibility is that information from each modality encoded in a motor-based frame of reference^51^ is used in judging congruence.

Second, biomechanical constraints also contribute to causal inference, as illusory postural displacement differed with biomechanical constraints between the posture of the actual hand and mirror-reflected hand, even when angular disparity and unimodal precision remained constant. In addition, sense of ownership differed between the 90° and 270° conditions, despite equal absolute angular disparity in these cases. How might biomechanical constraints be processed during visual-proprioceptive integration? Studies have shown that reaction times for judging the laterality of a hand image are affected by both angular disparity and biomechanical constraints between the viewer’s hand posture and the hand image^13–16, 18^. We hypothesize that participants used their body schema to implicitly rotate a representation of their actual hand to match the hand image–the motor simulation hypothesis. In our experiments, individuals may use some form of motor simulation to compare the visual and proprioceptive postural estimate.

We found that biomechanical constraints influence perceived hand posture across conditions. However, we only found that biomechanical constraints influenced ownership ratings when comparing performance in the 270°- versus 90°- less constrained conditions, but not when comparing less vs. more constrained conditions for 90° and 180°. One potential interpretation is that different patterns in the ownership versus postural displacement dependent variables reflect different processes, as seen in the rubber hand illusion^23, 45, 46^. Although this is possible, we do not yet want to make this claim. We note that the question of “ownership” in the rubber hand illusion is fairly clear, in that the baseline assumption is that the participant has never felt embodiment of the rubber hand before the illusion. Therefore, increases in ownership ratings reflect embodiment of the rubber hand caused by experimental manipulations. However, in the mirror box illusion, the mirror-reflection of their hand is more embodied than a rubber hand before illusion onset, given that it is controlled by the viewer and is their own hand. Therefore, questions about whether the mirror hand is “my own left hand” or “part of my body” could be answered in a similar manner across conditions. Given this, results from ownership questions may be noisier than those from postural displacement, perhaps leading to the non-significant result. Future research with more detailed ownership questionnaires and larger sample sizes can address this question.

Overall, we present a new illusion, in which viewing a hand making synchronous movements in opposing postures results in rotation of perceived hand posture towards the visual estimate. Critically, the amount of illusory displacement increased as the rotation from the proprioceptive to visual estimate became shorter and less biomechanically constrained. These results provide evidence that, in addition to principles of optimal weighting, biomechanical constraints from the body schema are processed in multisensory integration.

## Methods

### Experiment 1

#### Participants

In a pilot version of the experiment, we tested 24 participants and obtained acceptable power (1-β > 0.75) as well as effect size (Cohen’s d > 0.7). We therefore aimed at testing 24 participants. Twenty-five students (13 male, 12 female, ages 18–21, all right-handed), recruited from the General Psychology participant pool at the University of Delaware, took part in the experiment. One participant who was repeatedly observed not looking at the mirror hand was excluded from analysis, resulting in twenty-four participants (12 male, 12 female) included in the presented analysis. All studies were approved by the University of Delaware IRB. All participants signed informed consent forms before the experiment and received course credit as compensation. All methods were performed in accordance with the relevant guidelines and regulations.

#### Apparatus

The mirror box consisted of an acrylic mirror (16″ (40.6 cm) deep × 12″ (30.5 cm) tall) aligned with the participant’s midsagittal plane, mounted at the center of and perpendicular to a flat wooden base (36″ (91.4 cm) wide × 16″ (40.6 cm) deep). To block vision of the reflected upper arm and the body, two black curtains hung from rods on each side of the mirror box. To constrain participants’ hand posture, two wooden frames locked the participant’s wrists in the selected posture. The wooden frames were adjustable based on the size of each individual’s wrists and allowed only minimal wrist rotation once tightened. The wooden frames were placed 6″ to the left and right of the mirror, such that the two hands were equidistant (6″) from the mirror.

#### Design and Procedure

Experiment 1 was a 2 (postural congruence) × 2 (movement synchrony) × 2 (vision) within-subjects design (see Fig. 1). First, the hands were either in congruent postures (both palms facing down) or incongruent postures (left hand facing up, right hand facing down). Second, participants repetitively opened and closed their hands synchronously or asynchronously, with asynchronous movements creating a temporal and spatial conflict between the movement of the hidden hand and the mirror-reflected hand. Inputs from different modalities are more likely to be combined if they are spatially and/or temporally proximal–the spatial and temporal rules of multisensory integration^47, 48^. Therefore, we predicted increased multisensory integration when the unseen and mirror hand were in spatially congruent postures and during temporally synchronous movements. Finally, to exclude the possibility that simply making hand movements alone would cause changes in the perceived posture of the unseen hand, participants either viewed the hand reflection (mirror vision) or a black sheet that covered the reflective surface of the mirror (occluded vision). Each of the eight conditions was presented once in a random order for each individual. The length of each experiment was 30 minutes.

At the beginning of each trial, the participant placed his/her hands into the wooden frames in the selected postures. Then, the participant was instructed to open and close his/her hands for 30 seconds. In prior studies^38, 39^, participants made movements at 60 beats per minutes or slower. To make the visuomotor information more salient, participants in our study made movements to a metronome set at 70 beats per minute. Once 30 seconds elapsed, the metronome stopped and the participant briefly described their hand position verbally (verbal responses not analyzed) and was then provided with the following questions. First, the participant was presented with a paper-based circular posture scale to measure changes in the perceived posture of the unseen (left) hand (Fig. 3a). Participants were told that different positions on the circle represent different hand orientations, as marked by the hands along the circle. The task was to mark along the circle the perceived unseen hand posture. If participants did not fully understand, the experimenter would randomly pick a position along the circle and demonstrate the corresponding hand orientation. From this measure came our primary dependent variable for Experiment 1, postural displacement, defined as the difference in degrees between the participant’s response on the circular posture scale and the actual posture of their unseen left hand.

Next, the participant responded using a continuous visual analog scale (VAS, ranging from “completely disagree” to “completely agree”) to questions presented in random order using E-Prime (Psychology Software Tools, Inc, Pittsburgh, PA). One question was whether the unseen (left) hand was felt as in the same posture as the viewed hand in the mirror (the posture-matching question), whereas five other questions were presented to index perceived ownership of the viewed hand (see Supplementary Table S1 in the Supplemental Material available online). Four ownership questions were taken from prior studies^37, 41^, one question was newly added to ask whether movements on the viewed hand were felt as the movements on the unseen (left) hand. Cronbach’s alpha of the ownership questions was high (>0.85) across all experimental conditions, indicating high consistency among the questions. As these questions specifically referenced the viewed hand in the mirror, they were only asked on mirror vision trials. Results and analyses from these visual analog scale questions for both experiments are presented in the Supplemental Materials.

#### Statistical Analyses

Normality tests of residual errors were performed using the Shapiro-Wilk test in SPSS. Factorial analyses were performed with a permutation version of ANOVA, which does not make assumptions regarding the distribution of observed data, using the lmPerm R package (https://cran.r-project.org/web/packages/lmPerm/vignettes/lmPerm.pdf). In permutation tests, data points are reshuffled and randomly reassigned to experiment conditions. For example, data from subject X in condition A is reassigned to subject Y in condition B. In each permutation, *F-*values of the main effects and interactions of the resampled data are calculated. For each effect of interest, the *p*-value equals the percentage of permutations, out of all permutations, in which the *F-*value is equal to or larger than the actual observed *F*-value. Small *p*-values (e.g. 0.05) indicate that the observed effect ranks near the top among all the resampled effects, which in turn indicate that the observed effect is not a random event. After each permutation, the lmPerm package calculates the standard error of *p*-values obtained so far, and the permutation process terminated when the estimated standard error was below a criterion, i.e. when the *p*-values become stable (https://cran.r-project.org/web/packages/lmPerm/vignettes/lmPerm.pdf). The full model for objective ratings (i.e. postural displacement) contained the main effect of postural congruence (congruent, incongruent), movement synchrony (synchronous, asynchronous), and vision (mirror, occluded) as well as their interactions. Given that ownership questions were not given for the no mirror condition, the full model for ownership ratings contained the main effect of postural congruence and movement synchrony, along with its interactions. All post-hoc pairwise comparisons used permutation tests. Correlation analyses were performed with Spearman test in SPSS, with α = 0.05.

## Experiment 2

### Participants

As with Experiment 1, we aimed to test 24 subjects for each between-subjects manipulation. Prior studies have shown the effect of biomechanical constraints on the representation of both the left and right hand^13, 14^, we therefore wanted to ensure that our results generalize to both hands. Fifty-three new participants (30 male, 23 female, ages 18–23, all right-handed) from the General Psychology subject pool in the University of Delaware participated in Experiment 2. Twenty-six participants (17 male, 9 female) were tested with the left hand hidden behind the mirror (the left-hand group), and twenty-seven participants (13 male, 14 female) were tested with the right hand hidden behind the mirror (the right-hand group). Two participants in the left-hand group and four participants in the right-hand group were excluded from the final analysis due to excessive wrist rotation that moved the wrist constraint frame in one or more trials, resulting in twenty-four participants (16 male, 8 female) in the left-hand group and twenty-four participants (11 male, 13 female) in the right-hand group in the final analysis.

### Apparatus, Design and Procedure

This experiment examined the effects of angular disparity and biomechanical constraints on illusory displacement. Angular disparity is defined as the difference (in degrees) between the proprioceptively-defined and visually-defined hand posture in any condition. We varied the amount of angular disparity and biomechanical constraints in seven postural conditions, shown in Fig. 2. Each condition is named based on two components: angular disparity and the relative biomechanical constraints in moving from the proprioceptive estimate to the visual estimate of the hidden hand posture (i.e. the posture of the hand reflection). The 0°-no rotation condition (Fig. 2a) served as the control condition in which no illusory displacement was expected. Next, when angular disparity was 90° or 180°, pronation from the proprioceptive estimate to the visual estimate (Fig. 2b and d) involved *less* biomechanical constraints than supination (Fig. 2c and e) for both the left and right hand. This design allowed us to directly compare the amount of illusory displacement on trials matched for angular disparity, but differing in biomechanical constraints. The palm-outward posture in the 90°-less constrained (unseen outward) (Fig. 2f) and 270°-less constrained (unseen outward) (Fig. 2g) was made by clockwise rotation of the left hand (or counter-clockwise rotation the right hand) from palm down by 90°. The rationale for these conditions is presented in the results section. The length of each experiment was 30 minutes.

The same apparatus was used as in Experiment 1. To maximize multisensory integration, participants viewed the mirror hand and synchronously opened and closed both hands (Luria’s bimanual coordination task^40–42^ to a metronome set at 70 beats per minute in all trials. There were no asynchronous movement trials as in Experiment 1. For each participant, there was one trial per postural condition, with trial order randomized across subjects. Whether the hidden hand was the left or right hand was a between-subjects factor. Otherwise, all other procedures and dependent variables collected in Experiment 1 were the same in Experiment 2.

### Statistical Analyses

Normality tests, factorial analyses and correlation analyses were performed in the same ways as Experiment 1. Comparisons of the observed data to zero were performed with a one-sample permutation *t*-test using the DAAG R package (http://cran.bic.nus.edu.sg/web/packages/DAAG/DAAG.pdf). In each permutation, data points are randomly assigned with signs and the mean of the new sample is compared with the actual observed mean. The *p*-value equals the percentage of permutations, out of all permutations, in which the new sample mean falls outside the range from the negative to positive actual observed mean.

## Electronic supplementary material

Supplementary Information
