# Supplementary material for: Influence of the Body Schema on Multisensory Integration: Evidence from the Mirror Box Illusion
Source: Sci Rep. 2017 Jul 11;7:5060. doi: 10.1038/s41598-017-04797-0 (PMC5506044; doi:10.1038/s41598-017-04797-0)
Supplement: Supplementary file 1 — Supplementary Information [file 41598_2017_4797_MOESM1_ESM.pdf]

Influence of the Body Schema on Multisensory Integration: Evidence from the Mirror Box Illusion

Yuqi Liu, Jared Medina, Ph.D

Department of Psychological and Brain Sciences, University of Delaware

Corresponding author and address:

Jared Medina
University of Delaware
105 The Green, Room 108
Newark, DE 19716
Phone: 302-831-8724
Fax: 302-831-3645
E-mail: jmedina@psych.udel.edu

Supplemental Materials

Presented in this supplemental section are the results and analyses for questionnaire responses (see Supplementary Table S1 for the questions) for both Experiments 1 and 2.

Supplementary Table S1.

The questionnaire used in Experiment 1 and Experiment 2.

**Ownership questions**
It felt as though the hand in the mirror is my left hand.
It seemed like the hand in the mirror was part of my body.
It seemed like I was looking directly at my own left hand.
It seemed like my left hand was in the same location as the hand in the mirror.
It felt like the movements of the fingers I viewed were the movements I felt on my left hand.
**Posture-matching question**
It felt as though my left hand was palm down/inward/up.

Results

Experiment 1

Posture-matching question

Three participants were excluded from this analysis due to data collection errors. As with posture displacement, we analyzed ratings on the posture-matching question with a permutation version of ANOVA using the lmPerm R package (Wheeler, 2010, https://cran.r-project.org/web/packages/lmPerm/vignettes/lmPerm.pdf). The rationale for using this method is presented in the Methods section in the main article.

The full model we tested contained main effects of posture congruence, movement synchrony, and vision and their interactions. Higher ratings indicated that the unseen (left) hand was perceived as in the same posture as the mirror/right hand. As predicted (see Supplementary Fig. S1), ratings were higher in the congruent condition (*M* = 97.83, *SD* = 4.45) compared to the incongruent condition (*M* = 15.14, *SD* = 14.06), leading to a main effect of posture congruence, *p*

$< .001$. In addition, synchronous movements resulted in higher ratings than asynchronous movements, which led to a main effect of movement synchrony (Synchronous: $M = 60.26$, $SD = 9.70$; Asynchronous: $M = 52.71$, $SD = 6.99$), $p = .013$. Importantly, difference between synchronous and asynchronous movements was significant only in the incongruent, mirror vision condition ($p = .001$, *p*s in other conditions > .25), leading to a three-way interaction between postural congruence, movement synchrony, and vision, $p = .01$. This result is consistent with results of the circular posture scale, and indicated that motoric and temporal synchrony could overcome spatial posture incongruence and facilitate multisensory integration.

We also found a main effect of vision (Mirror vision: $M = 62.75$, $SD = 14.00$; Occluded vision: $M = 50.22$, $SD = 1.95$), $p < .001$. The interaction of movement and posture, $p < .001$, and the interaction of movement and vision, $p < .001$, were significant, such that difference between synchronous and asynchronous movements was larger in the incongruent versus congruent posture condition, and in the mirror versus occluded vision condition. Finally, the interaction of posture and vision was significant, $p < .001$, such that the difference in the ratings between congruent and incongruent posture was larger in the occluded versus mirror vision condition.

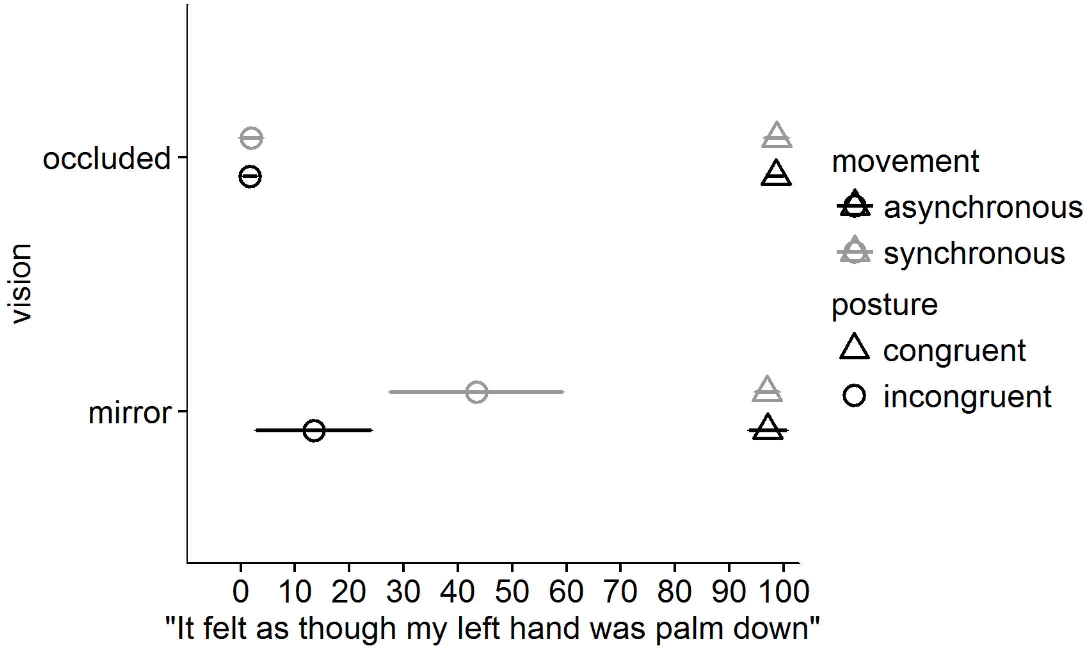

Supplementary Figure S1. Mean ratings on the posture-matching VAS question in Experiment 1. Whenever shown, error bars indicate within-subjects 95% confidence interval[1].

Experiment 2

Results

Postural Displacement Percentage

We tested the prediction that postural displacement in the 0˚-no rotation condition should not considerably differ from 0˚ with one-sample permutation *t*-tests using the DAAG R package (http://cran.bic.nus.edu.sg/web/packages/DAAG/DAAG.pdf). Postural displacement was not significantly different from 0˚ for the right hand (M = 0.21˚, SD = 10.69˚), p = .92, but was significantly greater than 0˚ for the left hand (M = 1.71˚, SD = 3.59˚), p = .042, which might reflect biases in the less biomechanically constrained direction in posture estimation.

Posture-matching question

One participant's response was excluded due to a data collection error. We performed a permutation test with angular disparity (90˚ and 180˚) and biomechanical constraints (less and more) as within-subjects factors, and hand (left and right hand) as a between-subjects factor. The dependent variable was responses on the posture-matching VAS question (Supplementary Fig. S2). Consistent with the circular response, ratings were significantly higher in the 90˚ (M = 62.55, SD = 26.65) versus 180˚ (M = 34.27, SD = 28.02) condition, $p < .001$. However, the effect of biomechanical constraints was not significant (less: M = 46.83, SD = 26.64; more: M = 49.99, SD = 26.44), $p = .347$. One possibility is that the more subjective nature of this question resulted in more variable responses that were not sensitive enough to reveal the influence of biomechanical constraints on multisensory integration. No interactions were significant (ps > .25).

We then compared ratings in 90˚- and 270˚-less constrained (unseen outward) to examine whether biomechanical constraints were taken into account in perceiving angular disparity (see Supplementary Fig. S2). We performed a permutation test with condition (90˚- and 270˚-less constrained (unseen outward) as the within-subjects factor and hidden hand (left and right hand) as the between-subjects factor. Ratings were significantly higher in the 90˚- (M = 69.68, SD = 29.01) than 270˚-less constrained (unseen outward) (M = 29.38, SD = 32.75), $p < .001$. No interactions were significant (ps > .25). These results indicated that although the absolute angular disparity was 90° in both conditions, angular disparity in the 270˚-less constrained (unseen outward) condition was perceived as 270° due to the effect of biomechanical constraints, which resulted in less multisensory integration.

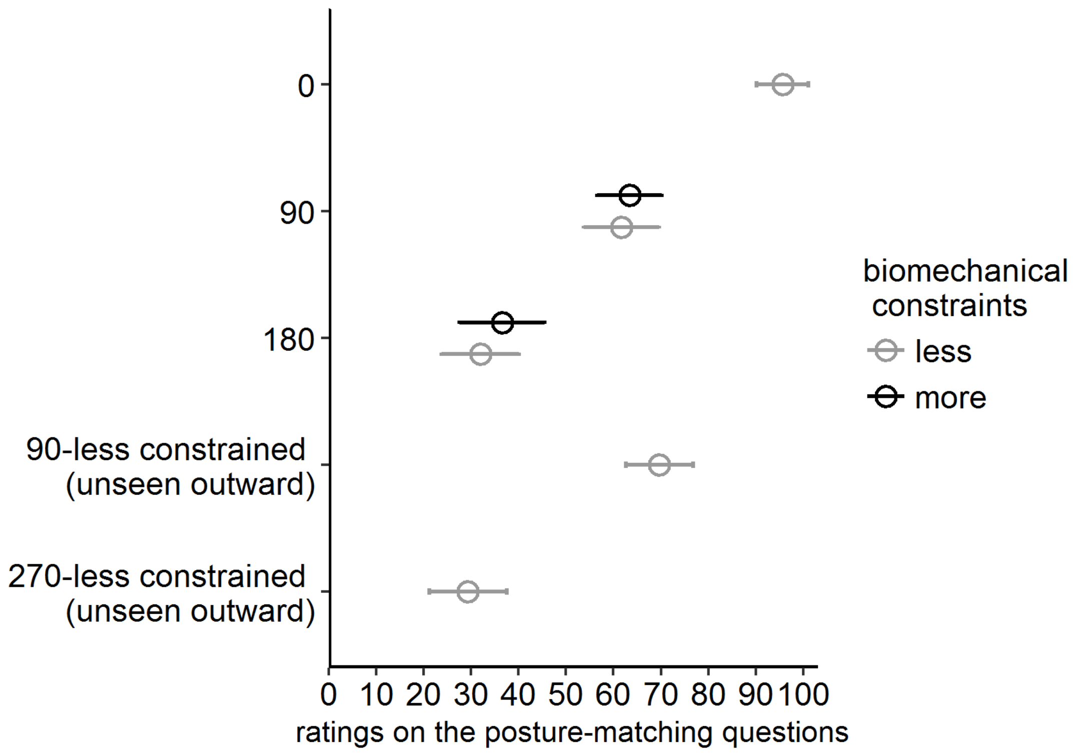

Supplementary Figure S2. Mean ratings on the posture-matching VAS question in Experiment 2, collapsed across the left and right hand.

In summary, we found the effect of angular disparity on perceived unseen hand posture. In addition, perceived postural difference was affected by biomechanical constraints, such that the 270˚-less constrained (unseen outward) condition resulted in less multisensory integration than the 90˚-less constrained (unseen outward) condition. Inconsistent with the circular posture scale response, biomechanical constraints had no effects on these subjective ratings. Given that we only have one question regarding the hand posture, and the subjective nature of this question, it is possible that variances in the ratings make this dependent variable not sensitive enough to reveal the influence of biomechanical constraints on multisensory integration.

References

1. Cousineau, D. (2005). Confidence intervals in within-subject designs: A simpler solution to Loftus and Masson's method. *Tutorials in quantitative methods for psychology*, *1(1)*, 42-45.
